# Investigation by Synchrotron Radiation Circular Dichroism of the Secondary Structure Evolution of Pepsin under Oxidative Environment

**DOI:** 10.3390/foods10050998

**Published:** 2021-05-02

**Authors:** Laetitia Théron, Aline Bonifacie, Jérémy Delabre, Thierry Sayd, Laurent Aubry, Philippe Gatellier, Christine Ravel, Christophe Chambon, Thierry Astruc, Jacques Rouel, Véronique Santé-Lhoutellier, Matthieu Réfrégiers, Frank Wien

**Affiliations:** 1 Institut National de Recherche Pour L’agriculture, L’alimentation et L’environnement (INRAE), UR370 Qualité des Produits Animaux, F-63122 Saint Genès-Champanelle, France; aline.bonifacie@inrae.fr (A.B.); thierry.sayd@inrae.fr (T.S.); laurent.aubry@inrae.fr (L.A.); philippe.gatellier@inrae.fr (P.G.); christine.ravel@inrae.fr (C.R.); thierry.astruc@inrae.fr (T.A.); jacques.rouel@inrae.fr (J.R.); veronique.sante-lhoutellier@inrae.fr (V.S.-L.); 2 Institut National de Recherche Pour L’agriculture, L’alimentation et L’environnement (INRAE), Plateforme D’exploration du Métabolisme Composante Protéomique (PFEMcp), F-63122 Saint Genès-Champanelle, France; jeremy.delabre43700@gmail.com (J.D.); christophe.chambon@inrae.fr (C.C.); 3 DISCO Beamline, Synchrotron SOLEIL, F-91192 Gif-sur-Yvette, France; matthieu.refregiers@synchrotron-soleil.fr (M.R.); frank.wien@synchrotron-soleil.fr (F.W.); 4 Centre de Biophysique Moléculaire, CNRS UPR 4301, Rue Charles Sadron, F-45071 Orléans, France

**Keywords:** protein oxidation, synchrotron radiation circular dichroism, digestion, pepsin, mass spectrometry

## Abstract

Food processing affects the structure and chemical state of proteins. In particular, protein oxidation occurs and may impair protein properties. These chemical reactions initiated during processing can develop during digestion. Indeed, the physicochemical conditions of the stomach (oxygen pressure, low pH) favor oxidation. In that respect, digestive proteases may be affected as well. Yet, very little is known about the link between endogenous oxidation of digestive enzymes, their potential denaturation, and, therefore, food protein digestibility. Thus, the objective of this study is to understand how oxidative chemical processes will impact the pepsin secondary structure and its hydrolytic activity. The folding and unfolding kinetics of pepsin under oxidative conditions was determined using Synchrotron Radiation Circular Dichroism. SRCD gave us the possibility to monitor the rapid kinetics of protein folding and unfolding in real-time, giving highly resolved spectral data. The proteolytic activity of control and oxidized pepsin was investigated by MALDI-TOF mass spectrometry on a meat protein model, the creatine kinase. MALDI-TOF MS allowed a rapid evaluation of the proteolytic activity through peptide fingerprint. This study opens up new perspectives by shifting the digestion paradigm taking into account the gastric digestive enzyme and its substrate.

## 1. Introduction

Food protein oxidation can occur during the whole processing axis, initiated during production, and developed during gastro-intestinal digestion [1]. Indeed, the physicochemical conditions of the digestive tract (oxygen pressure, low pH) were found to favor the meat protein chemical reactivity, in particular protein oxidation [2]. The implication of protein oxidation on meat digestion is of great importance, considering the consequences in terms of nutritional and health qualities [3]. In particular, protein oxidation, evaluated by carbonylation, was observed in the digestive tract, especially in the stomach, under its acidic conditions. Since food proteins are partly modified, digestive proteases, and in particular, pepsin, may be affected as well. Yet, to our knowledge, the link between endogenous oxidation and protein digestibility has only been studied from the substrate level. In particular, the biphasic relationship between food protein oxidation and digestibility is well known [4] through in vitro [5] and in vivo [6] studies. Shifting the digestibility paradigm is thus of great importance since the hydrolysis of proteins depends on the accessibility of the specific cleavage sites of digestive proteases but also on the modifications occurring on proteases. Because of the acidic pH of the gastric compartment, this proof-of-concept focuses on pepsin secondary structure evolution under oxidative conditions.

Therefore, the objective of this study is to determine the suitability of circular dichroism to monitor the pepsin folding and unfolding under an oxidative environment. The structural changes induced by external perturbations are directly linked to the circular dichroism spectra. Perturbation may come from thermal scans, pH, and, more generally, ionic concentration changes, small molecules, and ligand introduction, etc. Circular dichroism [7] is a method of choice for the dynamic follow-up of protein secondary and tertiary structures in solution. Its potential for quality control of gelatin from different batches has recently been demonstrated [8]. Synchrotron radiation circular dichroism (SRCD) was used to evaluate the impact of oxidation on the folding and unfolding kinetics of the pepsin. Using synchrotron radiation as a light source, acquisition of circular dichroism becomes faster [9] and reaches higher energies for better fold recognition [10]. The use of SRCD allows for the extension of the spectral domain down to 170 nm, increasing the information content of the spectral data. It allows distinguishing clearly the denaturation of the protein of interest and the autocatalysis of the enzyme. The modulation of the proteolytic activity of pepsin by oxidation was studied using a protein model, creatine kinase (CK). CK is a good substrate model for this study because it is one of the main cytoplasmic skeletal muscle proteins. The peptides resulting from its digestion by control and oxidized pepsin were then monitored by MALDI-TOF mass spectrometry.

## 2. Materials and Methods

### 2.1. Oxidation Induction of Pepsin

The chemical induction of pepsin oxidation was performed as follows: Pepsin was incubated for 2 h at 37 °C in a 20 mM phosphate buffer with oxidants (FeSO_4_/DETAPAC/H_2_O_2_)(DETAPAC: Diethylenetriaminepentaacetic acid) at various concentrations: 0.5, 1, and 3 mM. The acidification of gastric digestion was simulated by decreasing the pH during incubation: pH 5 at 0 min (T0), pH 4.5 at 30 min (T30), pH 3.5 at 60 min (T60), pH 3 at 90 min (T90), and pH 2 at 120 min (T120). An incubation control was obtained using only 20 mM phosphate buffer. Samples were taken at each time point, and each oxidation induction was performed in triplicate.

### 2.2. Creatine Kinase Hydrolysis

Proteolytic activity of pepsin and oxidized pepsin was evaluated using creatine kinase as a meat protein model. Creatine kinase was set at a concentration of 5 mg/mL, and pepsin, control, and oxidized as described in Section 2.1, was added with a ratio substrate/protease of 1/40. A hydrolysis control was obtained using only 20 mM phosphate buffer. Samples were taken at each time point, and each incubation was performed in triplicate.

### 2.3. Protein Oxidation Determination

Protein oxidation was measured by estimating the carbonyl groups using the method of [11] with slight modifications [12]. Carbonyl groups were detected by reactivity with 2,4 dinitrophenylhydrazine (DNPH) to form protein hydrazones. Absorbance at 370 nm was measured on a multiscan spectrum from Thermo Scientific (Waltham, MA, USA). The results were expressed as nanomoles of DNPH fixed per milligram of protein.

### 2.4. Synchrotron-Radiation Circular Dichroism

The kinetic of folding and unfolding of pepsin was measured by SRCD at the DISCO beamline of the SOLEIL Synchrotron (Gif-sur-Yvette, France). Thanks to synchrotron radiation, only 10 µL of pepsin at 5 mg/mL were deposited between 2 CaF_2_ coverslips with a guaranteed pathlength of 10 µm [13]. The radiation of DISCO beamline bending magnet was focused on the sample under nitrogen purge before collection by a photomultiplier tube locked to a photo-elastic modulator (Hinds) [14]. The beam size of 4 × 4 mm and the photon-fux per nm step of 2 × 10^10^ photons s^−1^ in the spectral band from 270–170 nm prevented radiation-induced damage [15].

Spectra were collected consecutively over time. Each spectrum was the mean of 3 accumulations. The buffer baseline was recorded sequentially and subtracted from the spectra before taking into account the concentration in residues. Estimation of the fold content of the proteins in solution was performed using BestSel [16].

### 2.5. MALDI-TOF Mass Spectrometry

For Matrix-Assisted Laser Dissociation-Ionization—Time-Of-Flight mass spectrometry ((MALDI-TOF MS) of creatine kinase digest, 1 μL of digest was manually spotted in triplicate on a polished steel target (MTP 384 Target Plate Polished Steel, Bruker Daltonics GmbH, Bremen, Germany). The matrix α-cyano-4-hydroxy-cinnamic acid (CHCA) was used at 7 mg/mL in water/acetonitrile 50:50 (*v/v*) with 0.2% trifluoroacetic acid, at a ratio of 1:1. Peptide spectra were acquired on an Autoflex Speed MALDI-TOF/TOF, equiped with a Smartbeam laser, using FlexControl software (Version 3.4; Bruker Daltonics GmbH, Bremen, Germany).

A total of 2500 spectra were accumulated randomly per sample, in triplicate for each technical triplicate. The laser power was constant for all the samples, and the laser focus was set at medium. Ion detection was done in reflectron mode at a mass range of m/z 750–3500. External calibration of spectra was done through the deposition of a peptide standard (Peptide Calibration Standard II, Bruker Daltonics) before each measurement on the same target.

### 2.6. Statistical Analysis

Values of carbonyl group content were mean ± standard error of 3 independent determinations. Values without common superscripts differ significantly (*p*-value < 0.001) between control and oxidized pepsin at each time-point, evaluated by t-test. The kinetic effect was evaluated for each condition, i.e., control and oxidized, by ANOVA (***: *p*-value < 0.001).

Principal component analysis (PCA) of MALDI-TOF spectra was calculated using ClinProTools software (Version 2.2, Bruker Daltonics). The spectra treatment consisted of the baseline subtraction, using the Top Hat algorithm with 10% of minimal baseline width, and smoothing, using the Savitsky–Golay algorithm with 2 cycles of 0.05 m/z width. The peak list was determined on the total average spectrum with a signal-to-noise of 3 and 5% of relative threshold base peak. Individual spectra were normalized using the TIC value.

## 3. Results and Discussion

### 3.1. Secondary Structure Evolution of Oxidized Pepsin Investigated by SRCD

**Figure 1:**
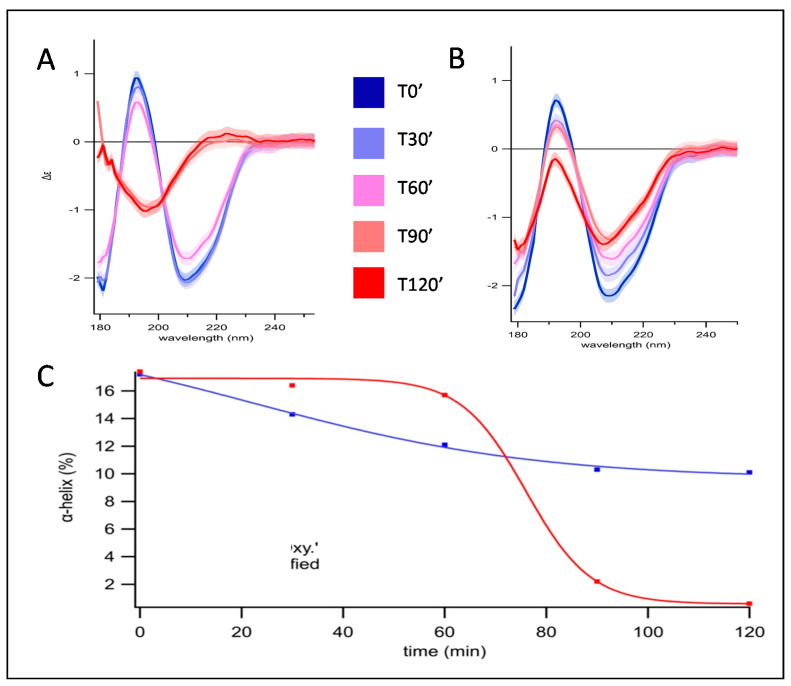
SRCD spectra of pepsin (**A**) and oxidized pepsin at 3 mM FeSO4/DETAPAC/H2O2 (**B**). The kinetic of gastric acidification was monitored, from 0 to 120 min. Spectra were recorded every 30 min and colored from dark blue, light blue, pink, orange, and dark red, respectively. For clearness, error contours at 1 sigma were drawn only for the first and last spectra, given their similarity for the other spectra. (**C**) Evolution of the percentage in α-helix in control (in red) and oxidized (in blue) pepsin during the kinetic of gastric acidification, from 0 to 120 min.

The secondary structure evolution of control and oxidized pepsin was monitored by SRCD (Figure 1A,B) from 0 to 120 min, corresponding to pH 5 to pH 2. Protein oxidation level was confirmed by the determination of carbonyl groups (Table 1) with a 5 to 6-fold difference between control and oxidized pepsin.

Pepsin, oxidized or not, did not present any strong structural changes between pH 5 and 3.5, from 0 to 60 min. After this point, the spectral analysis and the estimation of the folds content showed that control pepsin lost completely its alpha-helical content, compensated by an increase of beta-sheet structure (antiparallel relaxed +10%) and a slight increase of unstructured components (others) from 43% to 48%. After one hour, control pepsin denatured, corresponding to pH 3.5 (Figure 1C). From this point, the secondary structure was changing drastically in contrast to oxidized pepsin. After 60 min, the sinusoidal shape of the control spectra resembled spectra of polyproline II-type containing proteins (positive spectra values around 220 nm and negative values below 200 nm) [17], which was in accordance with the primary sequence (5% of proline content). Conversely, for oxidized pepsin, even at pH 2.5 or 2, the spectra were reduced in amplitude but conserved in shape. In addition, the limited loss of α-helical content (7%) indicated pepsin retained structure, in other words, it denatured less.

### 3.2. Proteolytic Activity of Oxidized Pepsin Investigated by MALDI-TOF Mass Spectrometry

**Figure 2:**
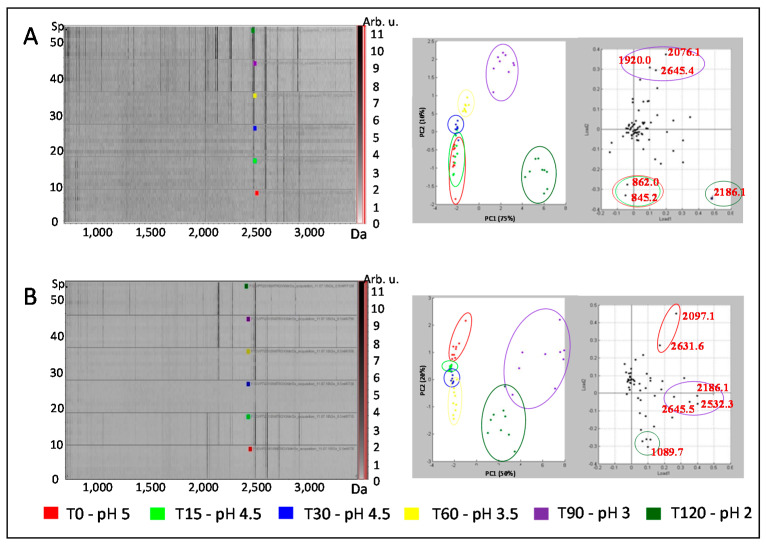
MALDI-TOF spectra of creatine kinase digestate by pepsin (**A**) and oxidized pepsin at 3 mM FeSO_4_/DETAPAC/H_2_O_2_ (**B**) monitored through the kinetic of gastric acidification, from 0 (in red) to 120 min (in dark green), to pH 5 to pH 2, respectively. MALDI-TOF individual spectra (*n* = 9 per each condition) are represented as a gel-view in the upper part. Principal component analysis of peptide spectra consists of the score plot on the upper part and the loading plot on the lower part.

The proteolytic activity of control and oxidized pepsin was evaluated using CK as substrate, and the resulting peptides were analyzed using MALDI-TOF mass spectrometry. The gel view, where each line is a spectrum and the peak intensities are indicated by grey level, gives a visualization of the hydrolysis kinetic of control and oxidized pepsin (Figure 2A,B).

The peptides spectra resulting from the hydrolytic activity of pepsin on CK showed a progressive profile with the appearance of a lower molecular weight peak, below 2500 Da, all along the hydrolysis kinetic, from the bottom (at T0, in red) to the top (at T120, in green) (Figure 2A). This evolution was confirmed by the PCA where the first two principal component (PC) supported 75% and 10% of variance, respectively. The PC1 allowed to distinguish the beginning of the kinetic (T0 to T30 mingled, associated with the m/z 845.2 and m/z 862.0) to the following time-point (T60, T90, and T120, associated with the m/z 1920.0, m/z 2076.1, m/z 2645.4, and m/z 2186.1 at T120). Even though the first two time-points were co-projected, the PC2 allowed us to distinguish the beginning of the kinetic better. The progressive profile of peptides spectra engendered the dynamics of CK hydrolysis by pepsin, all along the kinetic, i.e., from T0 to T120. The peptides spectra from the hydrolytic activity of oxidized pepsin on CK revealed a different progression during the kinetic (Figure 2B). Indeed, more peaks were detected at the beginning of the kinetic, above 2500 Da in particular, when compared to the results from control pepsin. This evolution was visualized by the spectra projection on PCA, where 50% and 20% of variance were supported by PC1 and PC2, respectively. The PC1 distinguished the end of the kinetic, T90 and T120, from the beginning that was clearly distinguished on PC1 (from T0 to T60). The beginning of the kinetic was associated with higher masses (m/z 2097.1 and m/z 2631.6 at T0 and m/z 2076.1, m/z 2645.4, and m/z 2948.9 at T90) than the end (m/z 1089.7 at T120). The differences observed at the beginning of the incubation of CK with oxidized pepsin were no longer seen at the end: The peptide spectra have the same profile as the one resulting from the action of control pepsin on CK. Therefore, the differences observed in peptide spectra resulting from the hydrolysis of oxidized pepsin were compensated during the 2 h incubation and digestion completed to the same level.

## 4. Discussion

This study aimed at elucidating how the oxidative conditions may impact the pepsin folding and unfolding in acidic conditions, and thus its hydrolytic activity, in an in vitro model representative of the gastric compartment. Starting the study at pH 5 mimics what happens after meat ingestion, where the buffering capacity of proteins will raise the gastric pH up to 5, as demonstrated in vivo [18,19,20]. As a consequence, the physicochemical environment of pepsin already activated at lower pH and present in the stomach will change and rise to pH 5. It takes approximately 2 h to retrieve the basal value of pH 2 [21].

Structural changes of pepsin do occur based on the evidence of secondary structure loss followed by very distinct kinetics. Indeed, control pepsin is very sensitive to pH resulting in the sudden collapse of helicity below pH 3.5, while its oxidized form seemingly preserved enough helicity and functional structural elements. The MALDI-TOF analysis of peptides resulting from control and oxidized pepsin hydrolysis also showed different patterns correlating the secondary structure decay. For control pepsin, a continuous progression during the 2 h digestion was observed. On the other hand, pepsin oxidation led to higher peak detection at the beginning of the kinetic (pH 5), which was compensated at the end, around pH 2.5, closer to the optimum enzyme pH. Similar observations were made after periodate oxidation, where the optimum pH for proteolytic activity of pepsin shifted toward a more neutral pH [22]. Oxidation has a protective effect on pepsin, preventing denaturation and preserving enzyme activity. This was demonstrated previously on myofibrillar proteins as substrates in the same conditions of 3 mM oxidizing agent [4]. Indeed, the catalytic sites of pepsin consist of residues Asp32 and Asp215 [23] protected inside a β-hairpin loop [24], which is less affected by denaturation (autodigestion) as opposed to the α-helix. To the best of our knowledge, no information is available regarding the digestive enzyme behavior under oxidation rendering a hypothesis difficult at this point regarding the mechanisms at stake. Nevertheless, circular dichroism spectral changes based on pepsin structure alteration revealed differences between oxidized and un-oxidized pepsin at low pH. These findings open new perspectives for enzyme behavior in digestion studies.

## 5. Conclusions

This proof of concept demonstrated the potential of synchrotron radiation circular dichroism for enzymatic digestion follow-up. The monitoring of folding and unfolding of pepsin was successfully achieved under an oxidative environment. The results highlighted the importance of the physicochemical conditions of the gastric environment on pepsin proteolytic activity. These findings support the idea of shifting the digestibility paradigm by focusing on digestive protease.

## Figures and Tables

**Table 1:** Carbonyl groups content in pepsin and oxidized pepsin during the kinetic of gastric acidification.

Kinetic, in Minutes		0	30	60	90	120
pH		5	4.5	3.5	2.5	2
Carbonyl group (nmoles DTNP/mg of protein)	Control	3.20 ± 1.03 ^a^	2.56 ± 0.79 ^a^	2.57 ± 0.74 ^a^	1.99 ± 0.21 ^a^	2.04 ± 0.22 ^a^
	Oxidized	15.43 ± 0.54 ^b^	14.95 ± 0.66 ^b^	14.95 ± 0.89 ^b^	14.75 ± 0.34 ^b^	13.98 ± 0.44 ^b^

The contents of carbonyl groups are expressed as nanomoles of DTNP bound per milligram of protein. Values are mean ± standard error of 3 independent determinations. Values without common superscripts differ significantly (^a^ and ^b^; *p*-value < 0.001) between control and oxidized pepsin at 3 mM FeSO_4_/DETAPAC/H_2_O_2_.
